# Intelligent fault diagnosis based on multi-source information fusion and attention-enhanced networks

**DOI:** 10.1038/s41598-025-20231-2

**Published:** 2025-10-16

**Authors:** Chuan Tong, Ling Chen, Jingzhe Zhang, Zhuowen Zhao

**Affiliations:** Kunming University, College of Information Engineering, Kunming, 650000 China

**Keywords:** Engineering, Mathematics and computing

## Abstract

Deep learning has been widely applied in the field of intelligent fault diagnosis, achieving remarkable progress in feature extraction and classification performance. However, most existing methods still face challenges in simultaneously capturing the temporal information and global features during the bearing operation process, which leads to insufficient acquisition of fault-related information. Moreover, under complex and harsh working environments, single-source fault diagnosis methods often struggle to stably extract fault features. To address these issues, this paper proposes an intelligent fault diagnosis method based on multi-source information fusion, aiming to enhance stability through the extraction and integration of rich feature representations. Specifically, vibration and current signals are transformed from raw time-domain data into time-frequency representations using continuous wavelet transform. At the image level, a grayscale-weighted fusion strategy is employed to effectively integrate multi-source information. In terms of model design, a diagnostic framework combining convolutional neural networks with attention mechanisms is constructed, enabling effective capture of both temporal information and global feature dependencies of bearing faults. Experimental results on a publicly available bearing fault dataset demonstrate that the proposed method consistently outperforms existing single-source and multi-source diagnosis models across various evaluation metrics, achieving higher fault recognition accuracy.

## Introduction

Mechanical components such as bearings and gears, as the core elements of modern industrial systems, are playing increasingly critical roles in heavy engineering fields such as oil extraction, mining development, and large-scale construction. The stable and efficient operation of mechanical equipment is not only essential for the smooth progress of industrial production, but also serves as a fundamental guarantee for economic development and social safety^1,2^.However, failures of mechanical components are the primary cause of mechanical equipment malfunctions. To ensure the long-term, reliable, safe, and efficient operation of such equipment and to promote the stable development of the manufacturing industry, it is essential to conduct in-depth research and exploration into high-precision and high-efficiency fault diagnosis technologies for mechanical systems.

Traditional machine learning methods for fault diagnosis primarily rely on expert knowledge and experience, where appropriate signal processing techniques are first selected, followed by the extraction and identification of fault features through manually defined thresholds^3^.However, due to limitations such as human subjectivity and the inability to efficiently handle large-scale data, traditional fault diagnosis models have struggled to meet the performance demands of modern industry. With the rise of deep learning technologies and the advent of the Industry 4.0 era, an increasing number of researchers have turned to intelligent diagnostic methods for the automatic extraction, selection, and classification of fault features in mechanical equipment. Currently, widely used deep learning-based diagnostic approaches include convolutional neural networks (CNNs), deep belief networks (DBNs), autoencoders (AEs), graph neural networks (GNNs), and long short-term memory networks (LSTMs). For instance, Shao et al. proposed an unsupervised domain-shared convolutional neural network for transfer fault diagnosis under variable-speed conditions, which significantly improved the model’s generalization ability across different operating scenarios^4^.Liu et al. addressed the strong temporal correlation characteristics of vibration signals by feeding raw vibration data directly into a convolutional neural network model with time-dislocation layers for motor fault diagnosis^5^. Wen et al. tackled the reliance on expert knowledge when converting signals into images by proposing a method that segments and stacks raw signals into matrices, which are then transformed into grayscale images and fed into a convolutional neural network for mechanical fault classification^6^. Jian et al. converted raw vibration signals from two sensors into Fourier coefficients and employed Dempster-Shafer evidence theory to fuse classification probabilities from four convolutional neural networks, thereby obtaining the final diagnostic result^7^. Yang et al. proposed a fault diagnosis framework for unseen operating conditions by jointly optimizing center loss and softmax loss to enhance feature discrimination^8^.

Zhang et al. employed the SLAP algorithm to optimize the parameters of deep belief networks, aiming to improve the accuracy and efficiency of bearing fault identification^9^. Tang et al. designed a novel adaptive convolutional neural network that utilizes acoustic images for fault recognition, enabling more precise fault diagnosis^10^. Yang et al. integrated an improved sparse autoencoder with a multi-level denoising strategy, effectively achieving early fault detection in mechanical equipment^11^. Wang et al. developed an extended deep belief network designed to more effectively extract useful information for detecting faults and anomalies in chemical processes^12^. Wang et al. also proposed an innovative BERT-BiLSTM-CRF model to efficiently extract key information from a constructed knowledge graph of power equipment faults^13^. Liu et al. constructed a spatiotemporal graph and utilized a graph neural network to extract critical feature information, thereby achieving effective diagnosis of rotating machinery faults^14^. Among these methods, convolutional neural networks (CNNs) have attracted considerable attention and recognition due to their outstanding performance in mechanical fault diagnosis through convolution and pooling operations. However, in real-world working environments, various noise interferences often obscure the periodic features of multi-source signals, posing challenges for CNNs in extracting effective local features. To overcome the limitations imposed by local receptive fields, some researchers have combined recurrent neural networks (RNNs) with other methods to enable global mining of fault information. Nevertheless, such approaches often result in more complex model structures, which can compromise diagnostic efficiency.

Based on the above considerations, this paper proposes a multi-source information fusion deep learning diagnostic model. By leveraging the Multi-Source Information Residual CNN-Attention(MIRCA) framework, the model learns more valuable fault information from multi-sensor data fusion and establishes a novel framework for bearing fault diagnosis. Specifically, we employ time–frequency methods to map one-dimensional signals from various sources into time–frequency representations. Through the integration of MIRCA with multi-sensor information fusion, the proposed framework comprehensively extracts informative features from multiple sources. The main contributions of this work are summarized as follows:

- The signals are preprocessed using Continuous Wavelet Transform (CWT) to capture richer information, and the resulting time-frequency representations are converted into grayscale images.
- A weighted fusion strategy is employed to obtain the optimal combination of information from multiple sources.
- By simultaneously incorporating Efficient Multi-Scale Attention (EMA) and Coordinate Attention (CA), the proposed model enhances its capability in multi-scale feature extraction and temporal information modeling, thereby enabling more effective extraction of multi-source information.

The structure of this paper is as follows: “Theoretical background” introduces the fundamental knowledge and theoretical framework, providing a detailed description of the workflow of the proposed method. Section “Methodology” presents an in-depth explanation of the proposed approach. Section “Experiments” discusses the case studies and evaluates the diagnostic performance of the proposed method across various fault diagnosis tasks. Finally, “Discussion” offers concluding remarks.

## Theoretical background

### Continuous wavelet transform

Continuous Wavelet Transform (CWT) is a method that projects a signal onto a set of wavelet functions with different scales and translations. It provides excellent time-frequency localization properties, making it well-suited for analyzing non-stationary signals. The formula for CWT is given by:

1 $$\begin{aligned} CW{T_f}(a,b) = [f(t),{\psi _{a,b}}(t)] = \frac{1}{{\sqrt{a} }}\int {f(t)\psi * (\frac{{t - b}}{a})} dt \end{aligned}$$

### Multi-source fusion strategy

**Figure 1:**
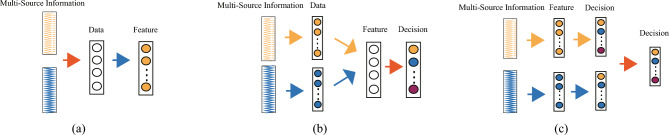
Multi-source fusion strategy.

Multi-source information fusion integrates data from multiple sources or sensors to obtain more accurate, robust, and comprehensive representations^15,16^. Researchers have developed the concept of multi-source information fusion to overcome the limitations of data from a single source or sensor. Each source or sensor may provide partial, noisy, or incomplete information; however, by combining them, the overall quality of information can be improved, enabling more informed decision-making^17^. Multi-source information fusion strategies can be categorized into three distinct types:

- Data-level fusion: This involves directly combining data from various sources to preserve all useful information, as illustrated in Fig. 1a.
- Feature-level fusion: This involves combining features extracted from different sources or sensors to create a unified feature representation, as shown in Fig. 1b.
- Decision-level fusion: This involves making final decisions or predictions based on the outputs from individual sources or sensors, as illustrated in Fig. 1c.

## Methodology

### Diagnosis steps

**Figure 2:**
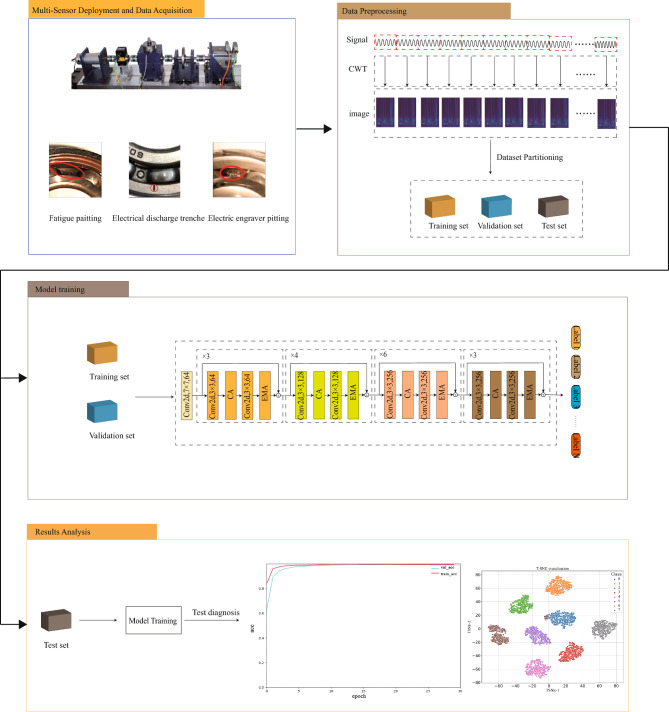
Diagnosis framework with MIRCA.

The implementation of the multi-source fault detection framework based on MIRCA is mainly divided into several modules, including data acquisition, image transformation, multi-source data fusion, feature extraction, and fault classification, as illustrated in Fig. 2. The overall diagnostic procedure is as follows:

- Motor bearing data are collected using acceleration and current sensors.
- The multi-source data are transformed into time-frequency images using CWT, and then divided into training, validation, and testing datasets.
- The transformed vibration and current time-frequency images are fused at the data level.
- The fused data are fed into the proposed MIRCA model, where convolution and attention mechanisms are used for feature extraction to train and validate the model.
- Multi-source test samples are used to evaluate the diagnostic performance of the proposed method.

### The proposed MIRCA model

**Figure 3:**
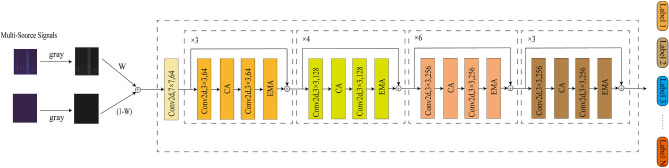
Model structure.

This section provides a detailed description of the main structure of the proposed MIRCA model. As illustrated in Fig. 3, the network architecture primarily consists of three components: a fusion module, a convolutional attention module, and a classification module.

### Multi-source fusion module

To fully exploit the complementary information provided by different types of sensors, the original signals, such as vibration and current signals, are first preprocessed and transformed into their corresponding time–frequency representations using CWT. Since signals of different types exhibit distinct characteristic patterns in the time–frequency domain, a unified image processing pipeline is required. To this end, the time–frequency images generated from each sensor channel are converted into grayscale images, highlighting the dominant energy distributions while reducing redundant information. This grayscale conversion facilitates subsequent image fusion operations. During the image fusion stage, a pixel-level weighted fusion strategy is employed to integrate multiple grayscale time–frequency images. The fusion weights are treated as learnable parameters and optimized during training, ensuring that the fused image preserves the key features from each source while enhancing both local details and global discriminative characteristics. The resulting fused time–frequency image not only encapsulates multi-perspective information that single-signal representations lack but also significantly improves the model’s capability to recognize and interpret multi-source information.

2 $$\begin{aligned} {F_3} = W{F_1} + (1 - W){F_2} \end{aligned}$$

where ${F_1}$ and ${F_2}$ represent the input grayscale images, *W* denotes the learnable weight parameter, and ${F_3}$ is the resulting fused image.

### MIRCA block

In bearing fault diagnosis scenarios, the raw vibration and current signals often exhibit non-stationary characteristics and multi-scale superposition, containing both global trend information and local impact features. To comprehensively extract these features, this paper designs a backbone network integrating convolution and attention mechanisms, referred to as the MIRCA model. The model balances the efficiency of convolution in local feature extraction with the attention mechanism’s capability to model long-range dependencies, thereby enabling precise capture of fault patterns from fused multi-source information. The proposed MIRCA model adopts a hierarchical progressive architecture, consisting of four stages in total. Each stage comprises two convolutional modules along with a CA module and an efficient EMA module, forming a unified unit for local feature extraction and global feature enhancement.

**Figure 4:**
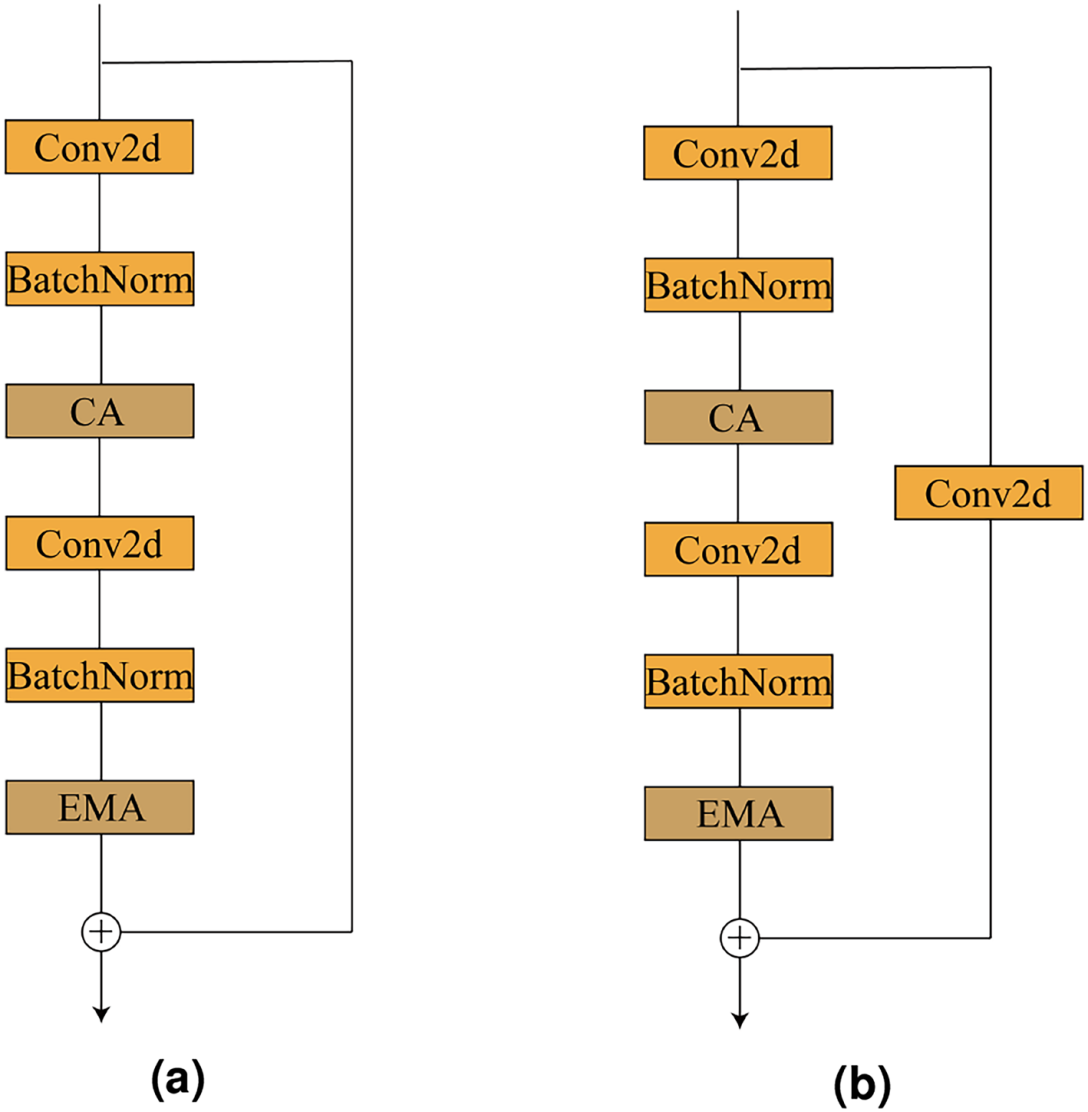
MIRCA block architecture.

To facilitate a deeper understanding of the structural design of the proposed network, this paper further elaborates on the construction details of the basic modules within each stage. As shown in Fig. 4a, the basic unit in the MIRCA model adopts a residual structure, consisting of a main branch and a shortcut branch.

In the main branch, the input features first sequentially pass through a convolutional layer followed by batch normalization, extracting local spatiotemporal features at different scales and thereby enhancing the model’s ability to capture fused multi-source information. The features then enter the CA module, which generates attention maps with directional awareness and positional sensitivity, enabling the model to more accurately locate and identify regions of interest. Subsequently, the features undergo another convolutional layer and batch normalization. The resulting features are fed into the EMA module, which possesses multi-scale perception and cross-spatial feature integration capabilities. This module models long-range dependencies between channels with relatively low computational cost and dynamically weights important regions, further improving the model’s sensitivity and discriminative ability for bearing faults. Notably, starting from the second stage, to accommodate the progressive increase in feature map size and semantic level, the first residual block of each stage introduces a convolutional layer with downsampling on the shortcut path, reducing spatial resolution while doubling the number of channels. As illustrated in Fig. 4b, this design enlarges the model’s receptive field.

**Figure 5:**
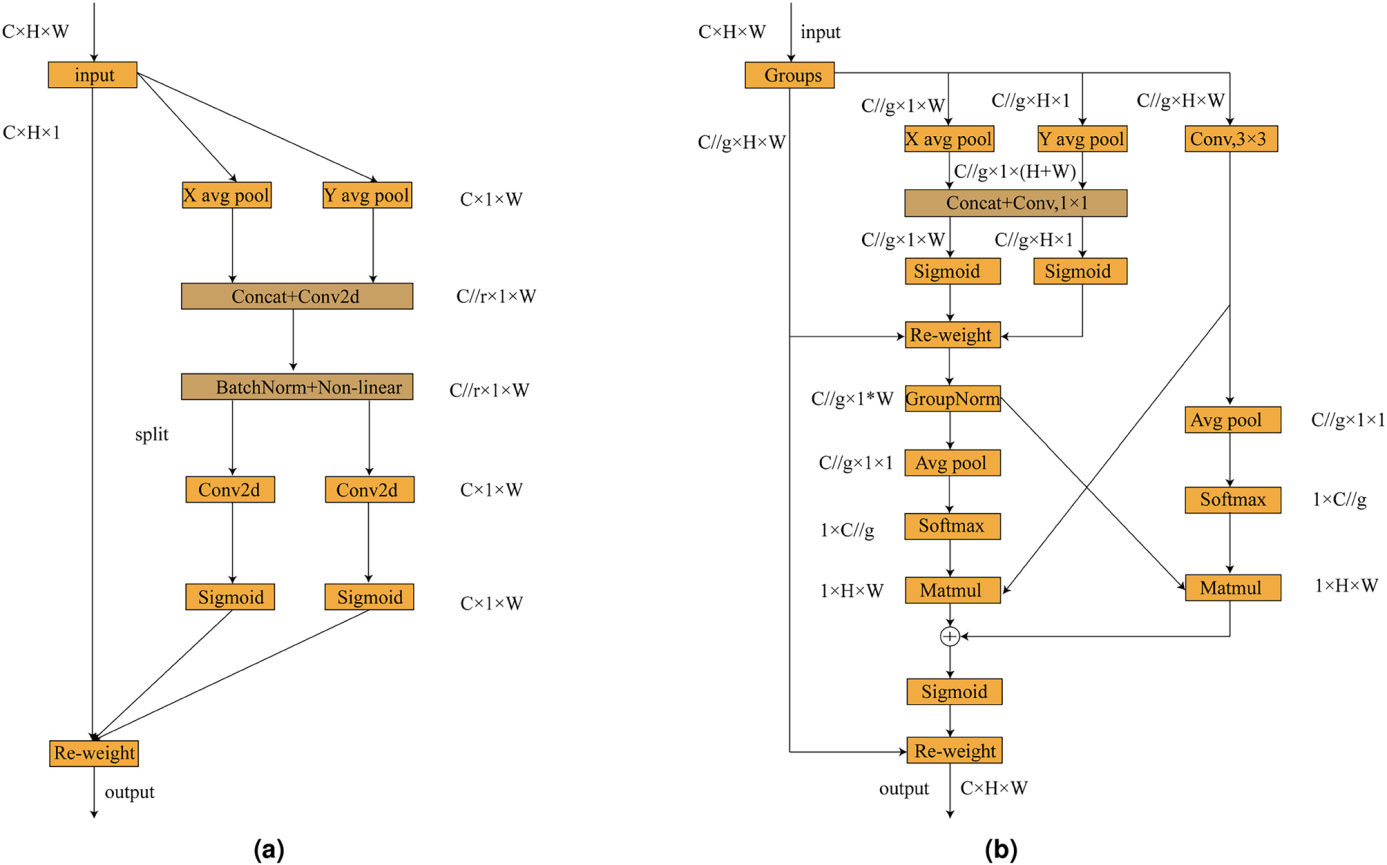
CA and EMA structures.

#### CA

This section presents the structural design of CA, as illustrated in Fig. 5a. CA is a lightweight and efficient attention mechanism designed to enhance a neural network’s ability to focus on important feature regions while preserving precise spatial location information.The core idea is to decompose the traditional 2D global pooling operation into two direction-aware 1D global pooling operations, which aggregate feature information along the horizontal and vertical directions, respectively. This enables the generation of direction-sensitive attention representations.

Specifically, given an input feature map ${\textrm{X}} \in {R^{C \times H \times W}}$, CA first performs two separate one-dimensional global pooling operations for each channel: one along the horizontal direction (vertical pooling), denoted as $z_c^h(h) = \frac{1}{w}\sum \limits _{i = 1}^W {x_c(h,j)}$, and the other along the vertical direction (horizontal pooling), denoted as $z_c^w(w) = \frac{1}{H}\sum \limits _{j = 1}^H {{x_c}(j,w)}$.These two aggregated features respectively encode the global contextual information of each channel in the vertical and horizontal directions. The results are then concatenated and passed through a shared $1 \times 1$ convolution layer, denoted as $f = \partial (Con{v_{1 \times 1}}([{z^h},{z^w}]))$.

This design enhances the model’s global capability in extracting fused multi-source information, enabling it to more effectively focus on global features along both the vertical and horizontal directions.

#### EMA

This section presents the structural design of the EMA module, as illustrated in Fig. 5b. In the EMA module, to more effectively capture the multi-semantic and multi-scale characteristics of bearing fault features, the input feature maps $\mathrm{{X}} \in {R^{C \times H \times W}}$,*X*are subjected to cross-channel dimensional grouping.Specifically, the EMA module divides the input feature map *X* into *G* sub-feature groups, each covering a portion of the channel dimension, with the aim of independently learning feature representations at different semantic levels. These groups are further represented by a set of descriptors $x=[x_{0}, x_{1},..., x_{G-1}],x_{i}\in R^{C//G\times H\times W}$. Through this grouping strategy, the network is able to focus on different types of information within each subspace.

To efficiently capture multi-scale spatial information, the EMA module introduces three parallel sub-network branches to generate attention weight descriptors for the grouped feature maps. This design is inspired by the integration mechanism of biological neurons, which can simultaneously process both local and global receptive fields. Such a structure enhances the model’s ability to perceive spatial information at different scales while maintaining parameter efficiency.Among these branches, two are $1 \times 1$ convolutional pathways, each incorporating one-dimensional global average pooling along a single spatial dimension. This operation enables compression encoding of channel information in the feature maps, facilitating lightweight modeling of cross-channel dependencies. The third branch employs a standard $3 \times 3$ convolutional kernel to introduce richer local contextual information, thereby enhancing the model’s capacity for multi-scale feature representation

In the EMA module, the two feature representations encoded along the height dimension of the image are first concatenated and then passed through a shared $1 \times 1$ convolution operation to model joint cross-channel representations. Unlike traditional attention mechanisms that often apply dimensionality reduction in the channel domain, no such reduction is performed in the $1 \times 1$ branches here, allowing more feature details to be preserved. The output of the $1 \times 1$ convolution is then factorized into two separate vectors, each of which is passed through a Sigmoid activation function to perform nonlinear fitting. This enables the model to capture potential two-dimensional binary-like distribution structures embedded in the convolutional outputs.

To enhance cross-channel feature interaction between the two $1 \times 1$ branches, the two channel attention maps within each sub-feature group are aggregated via element-wise multiplication, effectively fusing channel attention information from different perspectives. Meanwhile, the $3 \times 3$ branch employs a standard convolution operation to directly capture local spatial-channel interactions, thereby further expanding the expressive capacity of the features.

It should be noted that at this stage, two key tensors are retained: one from the $1 \times 1$ branch output and the other from the $3 \times 3$ branch output. To more effectively integrate global contextual information, a two-dimensional global average pooling operation is applied to the output of the $1\times 1$ branch prior to entering the joint channel feature activation mechanism. Specifically, the output of the $1 \times 1$ branch is directly reshaped to match the target channel dimension, denoted as $R_1^{1 \times C//G} \times R_3^{C//G \times HW}$. The two-dimensional global pooling operation is formulated as:

3 $$\begin{aligned} {y_c} = \frac{1}{{H \times W}}\sum \limits _j^H {\sum \limits _i^W {{x_c}(i,j)} } \end{aligned}$$

To further enhance multi-scale perception capability, spatial features from different branches are aggregated at the same processing stage to enrich information fusion along the spatial dimension. Meanwhile, to ensure consistency in the channel dimension before entering the joint channel feature activation mechanism, a two-dimensional global average pooling is also applied to the output of the $3\times 3$ branch to extract its global spatial semantics. The output of the $3 \times 3$ branch is directly reshaped to the target dimension $R_3^{1 \times C//G} \times R_1^{C//G \times HW}$ to match the structural requirements for fusion, thereby enabling efficient and accurate attention modeling.

## Experiments

**Figure 6:**
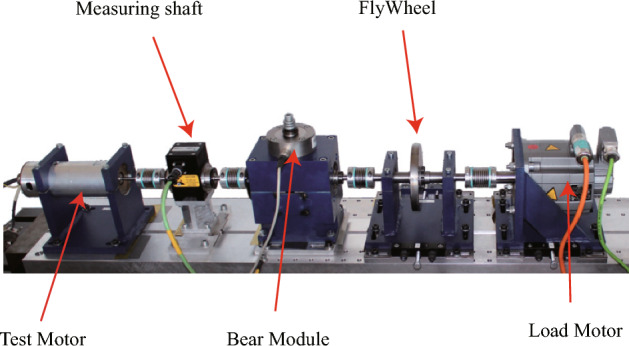
Schematic diagram of the lab bench.

**Figure 7:**
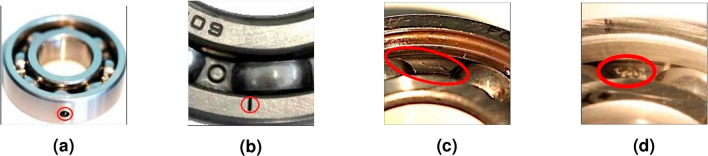
(**a**) Drilling holes; (**b**) Electrical discharge trenches; (**c**) Fatigue pitting; (**d**) Electric engraver pitting.

The Paderborn bearing dataset is provided by the KAT Data Center at Paderborn University^18^. The experimental setup is illustrated in Fig. 6. A current sensor and a piezoelectric accelerometer are employed to collect the bearing’s current and vibration signals, respectively, with a sampling frequency of 64 kHz. The experiments cover bearings under different operating conditions and various health states. Bearing faults are categorized into two types: artificially induced faults and naturally evolved faults generated through accelerated life testing. The naturally evolved faults are mainly manifested as fatigue spalls, while the artificial faults are introduced using drilling, electrical discharge machining (EDM), and manual electric engraving (MEE). The specific forms of these faults are shown in Fig. 7a–d.

Notably, the operating conditions in this case study include a rotational speed of 1500 rpm, a load of 0.7 Nm, and a force of 1000 N. The detailed description of the dataset is provided in Table 1.

**Table 1:** Descriptions of the Paderborn multi-source information dataset.

Operating conditions	Bearing health status		Label
1500 rpm, 0.7 Nm, 1000 N	Fault Types	Location	
	Electrical discharge trenches	Inner Race	0
	Electrical discharge trenches	Outer Race	1
	Fatigue pitting	Inner Race	2
	Fatigue pitting	Outer Race	3
	Drilling holes	Outer Race	4
	Electric engraver pitting	Inner Race	5
	Electric engraver pitting	Outer Race	6
	Noraml	/	7

### Experimental details

In the data processing stage, the collected vibration and current signals were first preprocessed using the Z-score normalization method. This step eliminates scale differences and mean shifts among different signal channels, thereby enhancing the model’s sensitivity to feature variations. Subsequently, the normalized multi-source signals were segmented into fixed-length sub-samples using a sliding window strategy with a window length of 1024. Among these sub-samples, 70% were allocated to the training set, 20% to the validation set, and the remaining 10% to the testing set.

### Comparative methods and implementation details

In this section, several state-of-the-art deep learning models commonly used for fault diagnosis were selected for comparison. The comparison focused on single-source signal methods, including:

Single-source information methods:

- CNN-LSTM.A fault diagnosis method combining a CNN-LSTM model with a sliding window strategy^19^.
- IFS-FACNN.An improved multi-scale convolutional neural network model^20^.
- SACL.A self-attention-based contrastive learning fault diagnosis method^21^.

Multi-source information methods:

- MH1DCNNs. Multi-head 1D CNNs are employed to capture valuable features from multi-source original signals, enabling effective fault diagnosis in motors^22^.
- M-IPISincNet.Based on an improved SincNet information convolutional network^23^.
- MSICNNs.A fault detection method based on an improved one-dimensional convolutional neural network and an improved two-dimensional convolutional neural network^24^.

The initial weights were randomly initialized, which means that the neural network’s starting state may vary with each training run, potentially causing variations in diagnostic results. To mitigate the impact of this randomness, all methods were configured with a uniform learning rate of 0.001 and employed the stochastic gradient descent (SGD) optimizer to minimize the cross-entropy loss function. The entire training process was implemented using PyTorch 1.8.0 and Python 3.8, and experiments were conducted on a system equipped with an NVIDIA GeForce RTX 4090 GPU.

**Table 2:** Diagnostic accuracy of different methods.

	Method	F1 score	Accuracy	Parameters	Inference time (ms)
Single-source information	CNN-LSTM	71.25%	71.55%	133k	4.26
	IFS-FACNN	89.32%	89.41%	79k	140.4
	SACL	97.05%	97.05%	133k	5.83
Multi-source information	MH1DCNNs	98.14%	98.14%	4199k	10.99
	M-IPISincNet	98.15%	98.25%	66k	59.99
	MSICNNs	99.08%	99.09%	5039k	1297.55
	MIRCA	99.61%	99.61%	21621k	27.62

As shown in Table 2, multi-source data fusion methods significantly outperform traditional single-source approaches in fault diagnosis, indicating that incorporating multi-source information enables the capture of more comprehensive feature representations. The proposed MIRCA method achieves superior performance across multiple evaluation metrics compared to existing single-source and multi-source fault diagnosis models. Specifically, MIRCA attains an F1 score and accuracy of 99.61%, which is notably higher than the best comparative method, MSICNNs, with 99.09%. Moreover, although MIRCA has a larger number of parameters (21.6M), its inference time is only 27.62 ms, far outperforming MSICNNs (1297.55 ms), thereby maintaining high efficiency. In summary, the proposed MIRCA method demonstrates excellent fault diagnosis capability.

**Figure 8:**
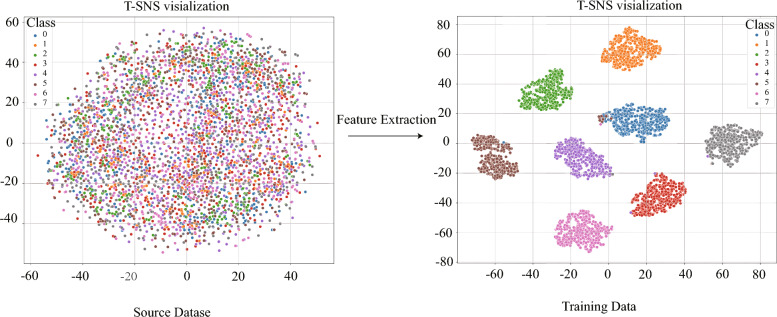
Feature visualization via the t-SNE of the proposed method.

### Visualization experiments

To demonstrate the effectiveness of fault diagnosis using the proposed MIRCA method and to directly illustrate the advantages of the multi-source information fusion strategy during feature extraction, t-distributed stochastic neighbor embedding (t-SNE) was employed to visualize the features extracted from the final hidden layer of the proposed method, as shown in Fig. 8.

As shown in the left half of Fig. 8, the two-dimensional features extracted from the original multi-source signals exhibit a relatively disordered distribution, with noticeable overlap among different classes, making clear separation difficult. In contrast, the right half of the figure displays the final features extracted by the proposed method, which are distinctly grouped into eight clusters in the two-dimensional space. The boundaries between categories are well-defined and the clusters are well-separated, demonstrating that the proposed method achieves superior discriminative power and representational capability in feature extraction, resulting in higher-quality features.

To comprehensively evaluate the performance of the proposed classification model in the fault diagnosis task, the confusion matrix was introduced as an assessment tool. The confusion matrix intuitively reflects the classification accuracy and types of errors for each category by comparing the predicted labels with the true labels. It is one of the important techniques for measuring the effectiveness of classification algorithms. Specifically, the horizontal axis of the confusion matrix represents the predicted label categories, while the vertical axis corresponds to the true labels of the samples. The values along the diagonal indicate the number of correctly classified samples—closer to the total sample count denotes better classification performance. Off-diagonal values represent the number of misclassified samples, which help further analyze the categories that the model tends to confuse.

**Table 3:** Ablation study of the MIRCA model.

Method	Accuracy	F1-Score
Baseline	85.32%	85.12%
Baseline+Ema	99.33%	99.33%
Baseline+CA	99.2%	99.12%
MIRCA	99.61%	99.61%

**Figure 9:**
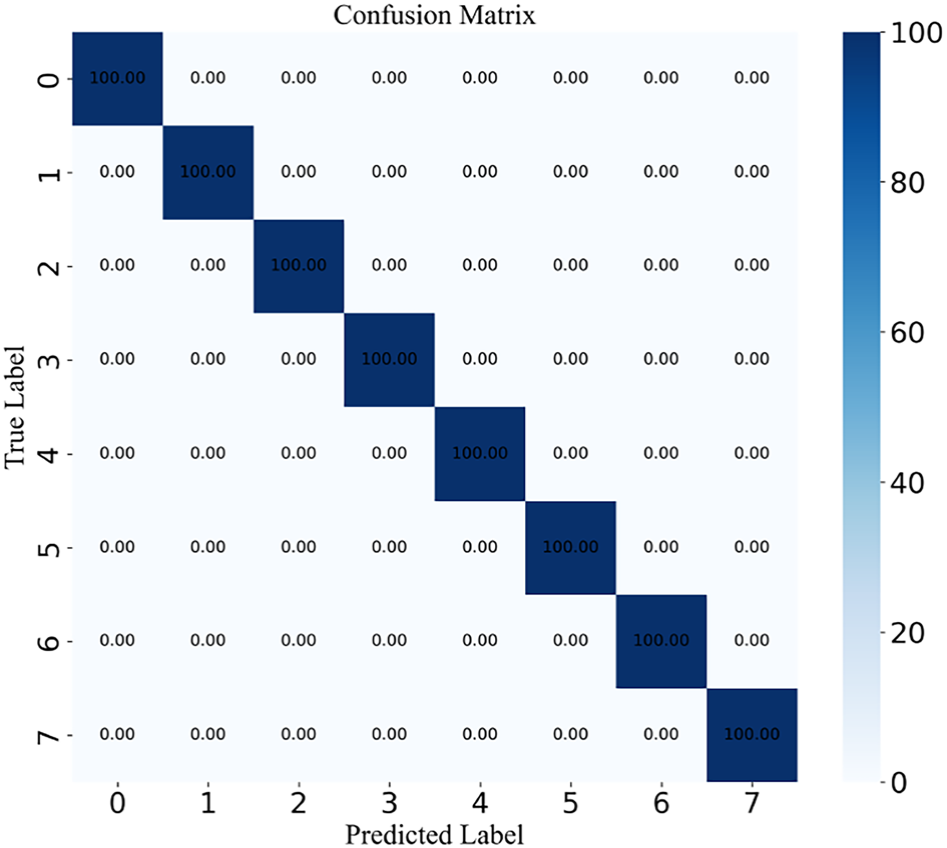
Confusion matrix of the proposed method.

Benefiting from its strong feature extraction and discriminative capabilities, the proposed MIRCA method demonstrates excellent performance in identifying eight health states of mechanical equipment in the dataset, as shown in Fig. 9. The confusion matrix results indicate a high consistency between predicted labels and true labels across all categories, with generally high classification accuracy and very few misclassified samples, suggesting that the model can effectively distinguish different types of fault features. The information conveyed by each confusion matrix is highly reliable, further validating the proposed method’s capability in fault feature extraction.

### Ablation study

To quantitatively evaluate the contribution of each key component in the proposed MIRCA framework, a comprehensive ablation study was conducted. The attention modules were systematically removed to assess their necessity and effectiveness in achieving superior diagnostic performance. Through systematic ablation experiments on the MIRCA model, we quantitatively evaluated the contribution of each key module to the overall diagnostic performance. As shown in Table 3, the baseline model (Baseline), without incorporating any attention mechanisms, achieves an accuracy and F1 score of approximately 85%, indicating a certain degree of feature extraction capability. After introducing the CA module individually, the model performance improves significantly, with accuracy and F1 score reaching 99.33% and 99.20%, respectively, demonstrating that both modules can effectively enhance the model’s perception of multi-scale features and spatial positional information. Finally, the full MIRCA framework, constructed by integrating EMA and CA simultaneously, achieves the best performance across all evaluation metrics (accuracy and F1 score both 99.61%), substantially outperforming configurations with either single attention module. These results validate the effectiveness of collaborative multi-attention mechanisms. In summary, the EMA and CA modules exhibit complementarity in enhancing the model’s perception and discrimination of multi-source fault features, and their combined use is key to achieving the superior diagnostic performance of the MIRCA model.

## Discussion

This thesis focuses on intelligent fault diagnosis of bearings based on multi-source information fusion and proposes a deep learning-based diagnostic method that enhances the model’s perception and recognition of fault features by integrating multi-source signals. The method jointly utilizes vibration and current signals and transforms the original time-domain signals into time–frequency representations via CWT. Subsequently, an efficient image-level fusion is achieved through a grayscale weighting strategy, which strengthens the discriminative capability of the extracted features. To validate the effectiveness of the proposed approach, experiments were conducted on the Paderborn multi-source bearing dataset, covering various operating conditions and fault types. The results demonstrate that the MIRCA model outperforms existing single-source and multi-source diagnostic methods, achieving an accuracy of 99.61% and superior F1 scores. Furthermore, t-SNE visualization and confusion matrix analyses confirm the method’s superiority in feature space separability.

In future work, further investigation is required to address the issue of information redundancy in information fusion. Additionally, as mechanical equipment often operates under variable and harsh conditions, it is necessary to incorporate transfer learning and enhance noise robustness. Finally, we aim to apply the experimental findings and theoretical insights to practical industrial environments.

## Data Availability

The data used in this study are available from the Paderborn University KAT Data Center at: https://mb.uni-paderborn.de/kat/forschung/kat-datacenter/bearing-datacenter.
